# Supplementary figures and images for: Grazing resistance developed in Escherichia coli K-12 during coexistence with a bacterivorous protist
Source: PLoS One. 2024 May 31;19(5):e0299885. doi: 10.1371/journal.pone.0299885 (PMC11142512; doi:10.1371/journal.pone.0299885)

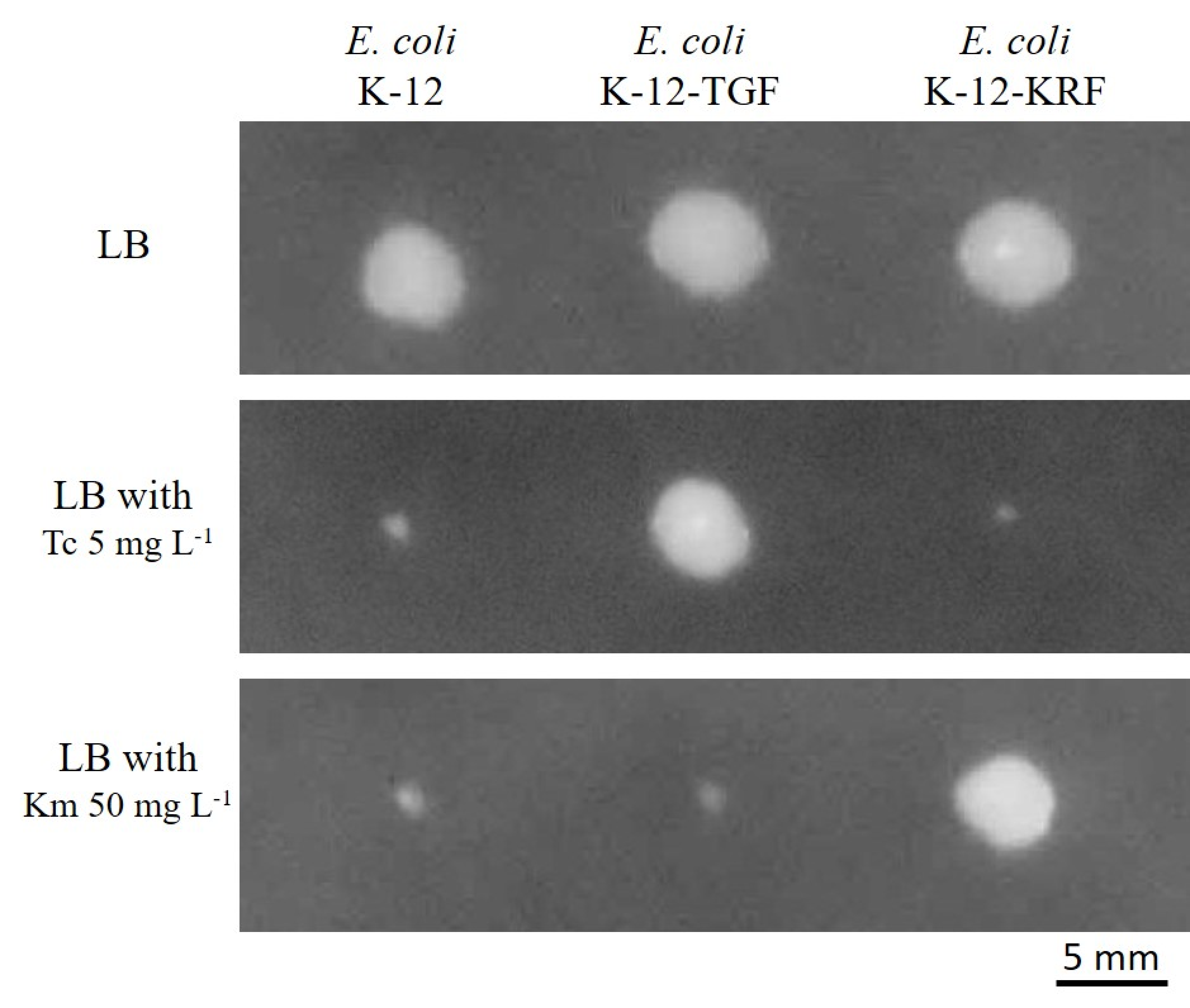

Supplement: S1 Fig — LB agar, LB agar containing 5 mg L-1 of Tc, and LB agar containing 50 mg L-1 of Km are used. The left colonies are E. coli K-12. The center colonies are E. coli K-12-TGF. The right colonies are E. coli K-12-KRF. All strains were transferred to medium and incubated overnight at 37°C. (TIF) [file pone.0299885.s001.tif]
