# Supplementary material for: Grazing resistance developed in Escherichia coli K-12 during coexistence with a bacterivorous protist
Source: PLoS One. 2024 May 31;19(5):e0299885. doi: 10.1371/journal.pone.0299885 (PMC11142512; doi:10.1371/journal.pone.0299885)
Supplement: S1 Table — The means and the standard deviations were calculated from these data. (PDF) [file pone.0299885.s002.pdf]

Original data in Table 2

Measurement Items		1st Reactor Eff	2nd Reactor Eff
pH	Day22	6.36	6.67
	Day25	6.40	6.75
	Day28	6.39	6.74
	Day31	6.45	6.76
All TOC (mg L^{-1})	Day22	22.36	20.45
	Day25	20.59	19.59
	Day28	19.95	19.71
	Day31	20.92	19.33
Supernatant TOC (mg L^{-1})	Day22	10.51	18.21
	Day25	9.64	17.74
	Day28	9.76	18.23
	Day31	9.84	16.45
Suspended TOC (mg L^{-1})	Day22	11.85	2.24
	Day25	10.95	1.85
	Day28	10.19	1.48
	Day31	11.08	2.88
Bacterial number (CFU mL^{-1})	Day22	3.77E+08	1.36E+06
	Day25	3.68E+08	8.55E+05
	Day28	3.43E+08	2.80E+06
	Day31	3.29E+08	2.56E+06
Protistan number (protist-cells mL^{-1})	Day22	-	8.03E+05
	Day25	-	7.75E+05
	Day28	-	8.72E+05
	Day31	-	9.75E+05
Conversion rate (CFU protist-$cell^{-1}$)	Day22	-	468
	Day25	-	474
	Day28	-	390
	Day31	-	335
