# Supplementary material for: Grazing resistance developed in Escherichia coli K-12 during coexistence with a bacterivorous protist
Source: PLoS One. 2024 May 31;19(5):e0299885. doi: 10.1371/journal.pone.0299885 (PMC11142512; doi:10.1371/journal.pone.0299885)
Supplement: S2 Table — The graph was made from these data. (PDF) [file pone.0299885.s003.pdf]

Numerical data of Fig 4 (A), (B), (C), (D) (CFU mL^{-1})

	Tube 1	Tube 2		Tube 3		Tube 4	
Days	*E. coli* K-12-TGF	*E. coli* K-12-KRF	*E. coli* K-12-TGF	*E. coli* K-12-KRF	*E. coli* K-12-TGF	*E. coli* K-12-KRF	*E. coli* K-12-TGF
0	2.42E+08	2.68E+08	-	2.68E+07	-	2.68E+06	-
7	1.97E+08	2.17E+07	-	3.60E+06	-	1.38E+06	-
14	1.59E+08	7.69E+06	-	3.12E+06	-	6.70E+05	-
21	1.45E+08	4.10E+06	-	2.95E+06	-	7.80E+05	-
28	1.28E+08	1.36E+06	4.00E+06	1.75E+06	4.00E+06	6.20E+05	4.00E+06
35	8.93E+07	9.88E+05	1.15E+04	7.00E+05	1.15E+04	8.77E+05	3.02E+04
42	9.85E+07	5.20E+05	2.65E+03	5.80E+05	2.60E+03	6.30E+05	1.45E+04
49	7.10E+07	7.50E+05	1.25E+03	3.45E+05	1.70E+03	1.16E+06	9.60E+03

Numerical data of Fig 4 (E), (F), (G), (H) (CFU mL^{-1})

	Tube 5	Tube 6		Tube 7		Tube 8	
Days	*E. coli* K-12-KRF	*E. coli* K-12-TGF	*E. coli* K-12-KRF	*E. coli* K-12-TGF	*E. coli* K-12-KRF	*E. coli* K-12-TGF	*E. coli* K-12-KRF
0	2.68E+08	2.42E+08	-	2.42E+07	-	2.42E+06	-
7	2.02E+08	4.20E+06	-	9.60E+06	-	8.20E+05	-
14	1.85E+08	4.15E+06	-	3.10E+06	-	5.10E+05	-
21	1.65E+08	1.06E+06	-	3.21E+06	-	5.40E+05	-
28	1.38E+08	1.14E+06	4.00E+06	1.36E+06	4.00E+06	7.65E+05	4.00E+06
35	1.21E+08	7.65E+05	2.30E+04	5.58E+05	3.80E+04	7.45E+05	3.06E+04
42	1.26E+08	4.50E+05	8.20E+03	1.90E+05	6.05E+03	5.60E+05	2.58E+04
49	1.12E+08	5.58E+05	1.50E+03	2.60E+05	3.45E+03	8.60E+05	3.31E+04
