# Supplementary material for: Grazing resistance developed in Escherichia coli K-12 during coexistence with a bacterivorous protist
Source: PLoS One. 2024 May 31;19(5):e0299885. doi: 10.1371/journal.pone.0299885 (PMC11142512; doi:10.1371/journal.pone.0299885)
Supplement: S3 Table — The graph was made from these data. (PDF) [file pone.0299885.s004.pdf]

Numerical data of Fig 5 (A) (CFU mL^{-1})

Days	1st Tube		2nd Tube		3rd Tube	
	E. coli K-12-TGF	*E. coli* K-12-KRF	*E. coli* K-12-TGF	*E. coli* K-12-KRF	*E. coli* K-12-TGF	*E. coli* K-12-KRF
0	1.11E+08	1.04E+08	1.11E+08	1.04E+08	1.11E+08	1.04E+08
7	6.50E+06	2.45E+06	4.75E+06	2.85E+06	6.60E+06	3.30E+06
14	7.85E+05	6.05E+05	4.50E+05	5.40E+05	5.30E+05	5.40E+05
21	1.29E+05	2.23E+05	1.06E+05	1.42E+05	1.01E+05	1.66E+05
28	1.23E+05	1.10E+05	4.33E+04	1.65E+05	5.67E+04	8.00E+04

Numerical data of Fig 5 (B) (CFU mL^{-1})

Days	1st Tube		2nd Tube		3rd Tube	
	E. coli K-12-TGF	*E. coli* K-12-KRF	*E. coli* K-12-TGF	*E. coli* K-12-KRF	*E. coli* K-12-TGF	*E. coli* K-12-KRF
0	2.80E+06	2.00E+06	2.80E+06	2.00E+06	2.80E+06	2.00E+06
7	4.60E+05	8.45E+04	1.70E+06	7.50E+04	8.25E+05	7.65E+04
14	2.70E+05	2.64E+04	3.03E+05	2.07E+04	2.07E+05	2.43E+04
21	3.95E+05	7.80E+03	2.25E+05	1.15E+04	2.32E+05	1.49E+04
28	4.55E+05	5.30E+03	2.10E+05	1.26E+04	2.40E+05	1.18E+04
