# Supplementary material for: Grazing resistance developed in Escherichia coli K-12 during coexistence with a bacterivorous protist
Source: PLoS One. 2024 May 31;19(5):e0299885. doi: 10.1371/journal.pone.0299885 (PMC11142512; doi:10.1371/journal.pone.0299885)
Supplement: S4 Table — The graph was made from these data. (PDF) [file pone.0299885.s005.pdf]

Numerical data of Fig 6: Apparent decrease rate constant k ($day^{-1}$)

Calculated by least squares

| | |
|---|---|
| Bar (1) | -0.024 |
| Bar (2) | -0.017 |

Average ± standard deviation

| | |
|---|---|
| Bar (3) | -0.042±0.074 |
| Bar (4) | -0.789±0.065 |
| Bar (5) | -0.063±0.051 |
| Bar (6) | -0.699±0.029 |
| Bar (7) | -0.168±0.076 |
| Bar (8) | -0.462±0.007 |
