# Supplementary material for: Grazing resistance developed in Escherichia coli K-12 during coexistence with a bacterivorous protist
Source: PLoS One. 2024 May 31;19(5):e0299885. doi: 10.1371/journal.pone.0299885 (PMC11142512; doi:10.1371/journal.pone.0299885)
Supplement: Attachment — Submitted filename: 2_Response to Reviewers.docx [file pone.0299885.s006.docx]

**Review Comments to the Author**

**Reviewer #1**: Dear Author

Thank you for your manuscript submission. Although this type of study is not new (there are a wide range of examples e.g., the bacteriovorous bacterium of Bdellovibrio etc.), it can be interesting in this field of research. There are two important points that should be included in the present manuscript:

1. It is necessary to add statistical analyses to show the efficacy of the obtained results through the statistical language.

(Answer 1)

Old Fig 4 was changed to new Fig 5. Statistical analyses were added (L303-325). Mainly, t-tests were conducted. The corrected parts are marked in red.

2. You have mentioned "two transformants were generated from E. coli K12 for grazing experiments. One was E. coli K-12-TGF, which possesses tetracycline resistance and green fluorescence. The other was E. coli K-12-KRF with kanamycin resistance and red fluorescence".

It is necessary to add the original figures relating to fluorescent colors (Red and Green) in this regard.

(Answer 2)

In RESULTS, “Prey bacteria” was added (L201-211). The corrected parts are marked in red. Also, microscopic pictures were added as new Fig 3.

3. It is important to interpret the statistical correlations in Discussion section.

(Answer 3)

A t-test was conducted, and the result was discussed (L337-339 and L365-368). The corrected parts are marked in red.

4. No effective conclusion has been presented.

(Answer 4)

The last part of the discussion was modified to show a conclusion (L402-414). The corrected parts are marked in red.

**Reviewer #2**: Your manuscript titled "Grazing resistance developed in Escherichia coli K-12 during coexistence with a bacterivorous protist" is a significant contribution to bioremediation processes. Your hypothesis is that indigenous bacteria can acquire some grazing-resistant traits by coexisting with protists in the natural environment. To verify the same you performed some experiments by using a bacterium, *Cupriavidus* sp. KN1 and a purified bacterivorous protist, *Spumella* sp. TGKK2

A development of grazing resistance in *Escherichia coli* K-12 was examined in the presence of a bacterivorous protist, *Spumella* sp. TGKK2. Two transformants were generated from E. coli K12 for grazing experiments. One was *E. coli* K-12-TGF, which possesses tetracycline resistance and green fluorescence and the other was *E. coli* K-12-KRF with kanamycin resistance and red fluorescence. First, you added protist-untouched *E. coli* K-12-KRF to protist-touched residual *E. coli* K-12-TGF that had been attacked by *Spumella* sp. TGKK2 in a batch test. Then the survivability of the respective strains was investigated. Consequently, *E. coli* K-12-KRF was predated preferentially. On the other hand, *E. coli* K-12-TGF in the same tube was less predated, indicating some grazing resistance. Also, a continuous culture device supplied with a glucose-containing medium as a substrate was operated. The device connected two complete mixed reactors in series. *E. coli* K-12-TGF was cultivated in the first reactor, and then grown *E. coli* K-12-TGF was predated by *Spumella* sp. TGKK2 in the second reactor. The effluent in the second reactor containing residual *E. coli* K-12-TGF and *Spumella* sp. TGKK2 was supplemented with batch-cultured *E. coli* K-12-KRF. Consequently, it was confirmed that batch-cultured *E. coli* K-12-KRF never exposed to protist was predated preferentially. These findings reveal that E. coli K12 acquires some predation resistance through coexistence with the bacterivorous protist.

The manuscript is correctly developed with a detailed description of the Materials and Methods applied, which allowed them to achieve robust results.

The only weakness that I would like to comment on is the lack of a conclusion after carrying out the present study since they only mention "However, the observed grazing resistance was not as significant as the level of predation resistance we observed in this research. More investigation is needed to explain the grazing resistance observed in this study."

(Answer 5)

The last part of the discussion was modified to show a conclusion (L405-414). The corrected parts are marked in red.

**Reviewer #3**: The research work sets clear objectives, the experiments seem solid, although there is doubt as to why the inoculum concentration is not standardized, it is not explained why the concentrations of 5 to 50 between *E coli* TGF and KRF.

(Answer 6)

Each of Tc and Km had its optimum concentration to distinguish *E. coli* TGF from KRF. The growth of these strains on LB containing 5 mg L^-1^ of Tc or 50 mg L^-1^ of Km was shown in S1 Fig., which was newly added.

The statistical results are expressed in the graphs, but are not described within the text.

If the reasons why it is not standardized and references are included, as well as the statistical data described in the text, the work will be even more robust.

(Answer 7)

Text data were added (L299-321). The corrected parts are marked in red. Also, raw data from Table 2, Fig 4, and Fig 5 were added as S1, S2, and S3 Table, respectively. These are cited in L219-20, L254, and L282-283. The data in Fig 6 can be calculated from these data, as explained in the manuscript.
